# Multiple Demographic, Lifestyle, and Biological Factors Associated With Brain Iron Deposition in the Basal Ganglia: A Comprehensive Analysis of 25,980 UK Biobank Participants

**DOI:** 10.1002/brb3.70862

**Published:** 2025-09-10

**Authors:** Pengcheng Liang, Linfeng Yang, Yiwen Chen, Zhenyu Cheng, Yian Gao, Chaofan Sui, Xinyue Zhang, Na Wang, Yuanyuan Wang, Changhu Liang, Lingfei Guo

**Affiliations:** ^1^ Key Laboratory of Endocrine Glucose & Lipids Metabolism and Brain Aging, Ministry of Education; Department of Radiology, Shandong Provincial Hospital Affiliated to Shandong First Medical University Jinan Shandong Province China; ^2^ Department of Radiology Jinan Maternity and Child Care Hospital Affiliated to Shandong First Medical University Jinan Shandong Province China; ^3^ Binzhou Medical University Yantai Shandong Province China

**Keywords:** basal ganglia, brain iron deposition, C‐reactive protein, lifestyle factors, quantitative susceptibility mapping, smoking, tea consumption, UK Biobank

## Abstract

**Background:**

The susceptibility values of the basal ganglia reflect the health status of these nuclei. We aimed to explore the associations between various demographic characteristics, lifestyle factors, and biological factors that have the potential to contribute to magnetic susceptibility and investigate the comprehensive impact of these multiple factors on basal ganglia susceptibility values.

**Methods:**

We included 25,980 participants from the UK Biobank. Linear regression analysis was employed to assess the relationship between basal ganglia susceptibility values and demographic characteristics (age, sex, ethnicity), lifestyle factors (tea consumption, coffee intake, smoking status, alcohol consumption, physical activity, insomnia status), and biological factors (C‐reactive protein, blood cell counts, anthropometric measures, blood pressure parameters).

**Results:**

Multiple factors demonstrated significant associations with basal ganglia iron deposition. Among biological factors, C‐reactive protein showed significant positive correlations with susceptibility values in the caudate nucleus (*β* = 0.028, *p* < 0.001), globus pallidus (*β* = 0.046, *p* < 0.001), and substantia nigra (*β* = 0.031, *p* < 0.001). Waist circumference, another biological measure, had substantial positive effects on most basal ganglia regions (*β* = 0.115 in caudate, *β* = 0.122 in putamen, *β* = 0.058 in globus pallidus). Among lifestyle factors, current smoking status was significantly associated with increased susceptibility values across all four basal ganglia nuclei (*β* = 0.053–0.061, all *p* < 0.001). Tea consumption demonstrated dose‐dependent protective effects, with daily consumption of ≥ 4 cups showing significant negative associations with all basal ganglia regions (−0.032 to −0.093 standard deviations). Age demonstrated significant positive associations with most basal ganglia regions. Gender differences were observed in tea consumption effects, with females showing stronger protective benefits (5.59 vs. 1.50 years of equivalent “rejuvenation” effect for 0–3 cups daily).

**Conclusions:**

We provide evidence for multiple demographic, lifestyle, and biological factors influencing brain iron deposition in healthy middle‐aged and elderly individuals. Systemic inflammation, smoking, and increased adiposity were associated with greater iron deposition, while tea consumption showed protective effects. These findings highlight potential targets for interventions aimed at maintaining brain health.

**Ethics approval and consent to participate:**

The UK Biobank protocol was approved by the NHS North West Multicentre Research Ethics Committee (21/NW/0157). All participants provided informed consent at recruitment, allowing for follow‐up using data linkage to health records.

## Graphical abstract

This graphical abstract illustrates the inverse relationship between daily tea consumption and standardized magnetic susceptibility in basal ganglia regions, with sex‐specific patterns observed in 25,980 UK Biobank participants.

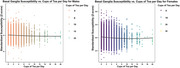

**Abbreviations**
ALS: amyotrophic lateral sclerosis
DBP: diastolic blood pressure
EGCG: epigallocatechin gallate
FA: Friedreich's ataxia
QSM: quantitative susceptibility mapping
SBP: systolic blood pressure

## 1 Background

The basal ganglia play a crucial role in motor control, learning, emotional regulation, and reward mechanisms, comprising a group of key subcortical nuclei (Arber and Costa 2022; Zhu et al. 2019). In a narrow sense, the basal ganglia include the caudate nucleus, putamen, substantia nigra, and globus pallidus. Pathological changes in the basal ganglia are central to neurodegenerative diseases (Trejo‐Lopez et al. 2022; Matuskey et al. 2020; van Bergen et al. 2016). Iron deposition in the basal ganglia is considered a biomarker for neurodegenerative conditions (van Bergen et al. 2016; Chen et al. 2019; Du et al. 2018; Huang et al. 2023). The accumulation of iron is a more sensitive indicator of neurodegenerative diseases than changes in the volume of gray matter (H. G. Kim et al. 2017). However, the relationship between iron deposition in the basal ganglia and multiple factors, including demographic characteristics, lifestyle factors, and biological markers such as inflammatory indicators, remains largely unexplored.

Iron deposition in the basal ganglia is closely associated with neurodegenerative diseases. Brain iron overload is a significant cause or contributing factor in various neurodegenerative conditions, including Alzheimer's disease, Parkinson's disease, Huntington's disease, Friedreich's ataxia (FA), and amyotrophic lateral sclerosis (ALS) (van Bergen et al. 2016; Chen et al. 2019; Huang et al. 2023; Dimov et al. 2023). Quantitative susceptibility mapping (QSM), an advanced magnetic resonance technique, quantifies local susceptibility changes in tissues influenced by iron content variations (Dimov et al. 2023; Y. Wang et al. 2017). In early Parkinson's disease, significant increases in susceptibility values are already observed in the putamen, globus pallidus, and substantia nigra (Chen et al. 2019). Iron accumulation in the substantia nigra plays a significant role in the pathophysiology of Parkinson's disease (Alushaj et al. 2024). Similarly, excessive iron accumulation in gray matter structures is present in Alzheimer's disease (AD), with significant increases in susceptibility values in the bilateral caudate nucleus and putamen of Alzheimer's patients (Huang et al. 2023). Additionally, the susceptibility value of the left caudate nucleus may serve as a biomarker for mild to moderate AD (Du et al. 2018). In early‐stage Huntington's disease patients without clinical symptoms, structures such as the caudate nucleus, globus pallidus, and putamen exhibit increased iron deposition, as evidenced by significantly elevated susceptibility values (van Bergen et al. 2016). Therefore, identifying more modifiable risk factors for changes in the susceptibility value of the basal ganglia and establishing preventive measures in the early stages are crucial.

The health of the basal ganglia is crucial for maintaining daily functions and quality of life, especially in the middle‐aged and elderly populations (Manza et al. 2015; Griffanti et al. 2018; Banerjee et al. 2022). Generally healthy individuals in these age groups may experience a range of age‐related changes, including mild cognitive decline and decreased motor abilities (van der Willik et al. 2021; Frolov et al. 2020). Paying attention to the brain health of this population, particularly the functional state of the basal ganglia, is important for enhancing their quality of life and preventing neurodegenerative diseases. In generally healthy middle‐aged and elderly individuals without apparent disease symptoms, changes in the susceptibility value of the basal ganglia may reflect disturbances in iron metabolism and early signs of neurodegenerative changes (Ndayisaba et al. 2019; Ravanfar et al. 2021). These changes may accumulate gradually without detection, ultimately impacting health and functionality.

While existing research has revealed the impacts of age and blood pressure on the susceptibility value of the basal ganglia, comprehensive studies examining the combined effects of demographic characteristics, lifestyle factors, and biological markers on brain iron deposition are relatively scarce (Q. Zhang et al. 2023; X. Li, Jin, et al. 2021). These lifestyle factors may play a significant role in the prevention and management of neurodegenerative diseases (De la Rosa et al. 2020; Kip and Parr‐Brownlie 2023; Popa‐Wagner et al. 2020; Santiago and Potashkin 2023). Tea, especially green and black tea, widely consumed globally, contains abundant bioactive components such as catechins and flavonoids, known for their notable antioxidant and anti‐inflammatory properties (Ohishi et al. 2016; Tipoe et al. 2007; Rha et al. 2019). Particularly, the polyphenolic compounds in green tea, such as EGCG (epigallocatechin gallate), have demonstrated potential neuroprotective effects in combating the neurodegenerative diseases AD and Parkinson's disease (Rha et al. 2019; Rezai‐Zadeh et al. 2005; Xu et al. 2020). Moreover, the association between habitual tea drinking and more efficient brain structures in the elderly further emphasizes the necessity to explore the relationship between tea intake and brain health (Xiang et al. 2024; Y. Zhang et al. 2021). Considering the crucial role of oxidative stress and brain inflammation in the disruption of brain iron metabolism, and the potential positive impact of tea consumption on brain health, investigating the relationship between tea intake and the susceptibility value of the basal ganglia becomes an important research topic (Y. Zhang et al. 2021; Mahoney‐Sánchez et al. 2021; Zucca et al. 2017; Teixeira Oliveira et al. 2023; S. Zhang et al. 2022).

With the intensification of population aging, brain aging and neurodegenerative diseases have become significant global challenges (Niccoli and Partridge 2012; Azam et al. 2021). The UK Biobank hosts the largest collection of high‐quality MRI brain scans, physiological data, and lifestyle factor measurements available today (Alfaro‐Almagro 2018). Our research examines the relationship between the susceptibility value of the basal ganglia and physiological data as well as lifestyle factors across a large population sample, promising new insights into slowing the brain aging process, reducing the risk of certain neurodegenerative diseases, and improving the management of brain functional disorders.

Building on previous research findings, we hypothesized that multiple demographic characteristics, lifestyle factors, and biological factors would show distinct associations with basal ganglia susceptibility values. The vast sample size available from the UK Biobank provides sufficient power to comprehensively assess the relative contributions of various factors including tea consumption, smoking status, inflammatory markers such as C‐reactive protein, and anthropometric measures in influencing brain iron deposition. Our study aims to provide a comprehensive evaluation of modifiable and non‐modifiable factors associated with basal ganglia health in a large, well‐characterized population.

## 2 Methods

### 2.1 Data Source and Study Population

The UK Biobank (www.ukbiobank.ac.uk) is a substantial prospective, observational cohort study, encompassing 502,269 individuals aged 45–69 years, aimed at exploring the impact of a wide range of exposures on health outcomes and disease development. Data including personal lifestyle, mental health, and health history details were collected from UK Biobank participants using questionnaires and interviews by trained staff. The study recruited people from all over the UK from 2006 for 5 years. A smaller group from these participants also had MRI scans and were part of our study from March 2014 to January 2018. The UK Biobank was approved by the North West Research Ethics Committee, and all participants signed informed consent.

The data quality control (QC) process of the UK Biobank has provided a highly reliable data source for biomedical research, particularly important in exploring complex issues related to human health. Our study leverages the high‐quality data from this database to focus on the association between the susceptibility values in the basal ganglia region (including the caudate nucleus, putamen, globus pallidus, and substantia nigra) and lifestyle factors in a generally healthy population. The basal ganglia, as key areas in the brain associated with motor control, cognition, and emotion, may reveal potential impacts of these lifestyle factors on brain health through changes in their susceptibility values. To ensure the accuracy and reliability of the research findings, strict participant selection criteria were implemented, including only individuals assessed as in good or excellent overall health status, to minimize interference from known pathological states. Moreover, to control for accuracy and avoid potential impacts of disease states on brain iron metabolism, individuals with histories of brain tumors, strokes, cerebral hemorrhages, nontraumatic intracranial hemorrhages, cerebral infarction, cerebrovascular diseases, multiple sclerosis, AD, Parkinson's disease, and cranial injuries were excluded. Detailed handling of missing values in the dataset ensured the integrity and accuracy of the analysis. Through rigorous data processing and QC measures, data from 25,980 participants were included in the study. This provides a solid data foundation for further exploring the relationship between susceptibility values in the basal ganglia and lifestyle factors.

In the scatter plot between the number of daily teacups consumed and the total susceptibility transfer in the basal ganglia region, we excluded individuals whose daily tea consumption was beyond 6.4 standard deviations (SD) from the mean. This stringent threshold was chosen to retain as much analyzable data as possible while eliminating significant outliers, resulting in the exclusion of 14 participants (nine males and five females). It is important to note that removing these outliers does not alter the significance or magnitude of the effects reported in our findings.

## 3 Measurements

### 3.1 Demographic Characteristics

Demographic data were collected during recruitment and included age (continuous variable, years, range 50–79), sex (male/female), and ethnicity (categorized as White, Asian, Black, Chinese, Mixed, and other).

### 3.2 Lifestyle Factors Assessment

Lifestyle factors were assessed through standardized questionnaires administered by trained staff. Dietary habits included tea intake and coffee intake (both measured in cups per day), with participants categorized based on daily consumption into three groups: 0–1 cups, 2–3 cups, and ≥ 4 cups per day. Substance use assessment covered smoking status (categorized as never, previous, current) and alcohol intake frequency, which was grouped as follows: (1) never/special occasions/1–3 times monthly, (2) once–twice weekly, and (3) three or more times weekly to daily. Sleep and stress indicators included insomnia status (categorized as usually, sometimes, rarely, never). Physical activity was measured as the duration of moderate activity in minutes per day.

### 3.3 Biological Factors Assessment

#### 3.3.1 Blood‐Derived Biomarkers

Biological samples were collected and analyzed for inflammatory markers including C‐reactive protein levels (mg/L) and hematological parameters including lymphocyte count, monocyte count, neutrophil count, and platelet count (all measured in 10^9^ cells/L).

#### 3.3.2 Anthropometric and Physiological Measurements

Physical examinations provided anthropometric data including body mass index (BMI, kg/m^2^) and waist circumference (cm), as well as cardiovascular parameters including systolic blood pressure (SBP) and diastolic blood pressure (DBP) (mmHg). Pulse pressure was calculated as the difference between SBP and DBP.

### 3.4 MRI Data Acquisition and Processing

Brain imaging data were obtained by using a 3.0‐T MRI imager (Siemens Skyra, Siemens Healthcare, Erlangen, Germany) with a standard 32‐channel radiofrequency receiver head coil. Susceptibility‐weighted MRI (swMRI) data were used for this study (3D GRE, TE1/TE2/TR = 9.4/20/27 ms, voxel size = 0.8 mm × 0.8 mm × 2.0 mm) as a measure sensitive to magnetic tissue constituents. QSM was derived from phase images captured from individual coil channels, which were then aggregated, masked, and phase‐unwrapped. The computation of magnetic susceptibility (*χ*) values involved a specialized QSM workflow that included the elimination of background fields, the inversion of magnetic dipoles, and calibration against cerebrospinal fluid (CSF), as outlined in previous studies. For each designated brain region, median *χ* values (expressed in parts per billion) were calculated across voxels, yielding a total of four distinct QSM imaging‐derived phenotypes (IDPs).

All UK Biobank brain MRI scans, including the multi‐echo GRE acquisitions used for QSM, undergo a rigorous automated QC pipeline, followed by visual inspection, to identify and exclude datasets with excessive head motion, poor brain extraction, or other acquisition artifacts before release (Alfaro‐Almagro 2018; Miller et al. 2016). Consequently, datasets available to researchers have passed these stringent QA checks, minimizing the likelihood that our findings are attributable to motion‐related artifacts, even among older participants who may move more during scanning. QSM reconstruction followed the established UK Biobank pipeline (C. Wang, Martins‐Bach, et al. 2022), which has been widely validated for robust susceptibility estimation in both research and clinical settings.

### 3.5 Statistical Analysis

We represented the overall characteristics using the mean (standard deviation) for continuous variables and proportions for categorical variables. We depicted the relationship between the average susceptibility values of the caudate nucleus, putamen, globus pallidus, and substantia nigra and age, as well as daily tea intake, in both males and females.

Participants were categorized based on their daily coffee intake into three groups: 0–1 cups, 2–3 cups, and ≥ 4 cups. Similarly, tea drinkers were classified into the same three categories based on their daily tea intake. To further examine the impact of insomnia, alcohol intake frequency, and smoking status on the susceptibility value of basal ganglia nuclei, participants were grouped according to their self‐assessed insomnia levels into “never/rarely,” “sometimes,” and “usually.” Smoking status was divided into “never,” “previous,” and “current.” For alcohol intake frequency, those who self‐reported as “never,” “only on special occasions,” or “one to three times a month” were grouped together; those reporting “once or twice a week” formed a second group; and those reporting “three or four times a week, daily or almost daily” were categorized into a third group.

We used linear regression to analyze the relationship between demographic characteristics (age, sex, ethnicity), lifestyle factors (tea intake, coffee intake, smoking status, alcohol consumption, insomnia status, physical activity), and biological factors (C‐reactive protein, waist circumference, blood pressure parameters, blood cell counts) with the susceptibility values of the caudate nucleus, putamen, globus pallidus, and substantia nigra. In our preliminary analysis of the average susceptibility values of the four basal ganglia nuclei (caudate, putamen, globus pallidus, substantia nigra) and the linear regression with log(1+ daily unit) of tea intake, considering the slight concavity in the LOWESS regression line between basal ganglia susceptibility values, daily tea intake, and age, we included linear and quadratic terms for daily tea intake, as well as interaction terms between tea intake and age and sex. Although the quadratic term for age was also included in the model, it is grouped under the vector of control variables for brevity. The full model is specified as follows:

Susceptibility value of BG = *β*_0_ + *β*_1_·*X_i_* + *β*_2_·*X_i_* ^2^ + *β*_3_·(*X_i_* × Sex*_i_*) + *β*_4_·(*X_i_* × Age_i_) + *γ*_1_·Age*_i_* + *γ*_2_·Age*_i_* ^2^ + *γ*_3_·Sex_i_ + *γ*_4_·Daily coffee intake*_i_* + *γ*_5_·Insomnia status*_i_* + *γ*_6_·Smoking status*_i_* + γ_7_·BMI*_i_* + *ε_i_*

Where *X_i_* is the standardized tea intake in log(1 + daily units), Age*_i_* is standardized age, and all continuous variables are standardized (*z*‐scores). Quadratic interaction terms (tea^2^ × age, tea^2^ × sex) were tested using an *F*‐test for nested models but did not provide a statistically significant or practically meaningful improvement in model fit; thus, only linear interaction terms were retained in the final model. We further used our regression model to calculate the change in the susceptibility values of the basal ganglia associated with an increase of three units in daily tea intake, stratified by gender. Our statistics were based on estimates from the full sample, controlling for variables such as daily coffee intake, insomnia status, smoking status, and BMI. We further benchmarked the predicted effects for an average 65‐year‐old UK Biobank participant against the effects associated with rejuvenation based on our regression model. All statistical analyses were performed using SPSS software (SPSS; version 26.0 for Windows). To assess whether the null findings related to coffee intake were due to insufficient statistical power, we performed a post hoc power analysis using the pwr package in R. Based on our sample size (4556 participants with ≥ 4 cups/day of coffee), assuming a small to moderate effect size (*f* ^2^ = 0.02–0.04) and *α* = 0.05 with 20 covariates in the model, the statistical power exceeded 99%. These results suggest that our analysis was sufficiently powered to detect biologically meaningful associations with coffee consumption. A *p*‐value of 0.05 was considered statistically significant, and *p*‐values were Bonferroni‐adjusted for multiple comparisons.

## 4 Results

### 4.1 Study Participants and Baseline Characteristics

**FIGURE 1:**
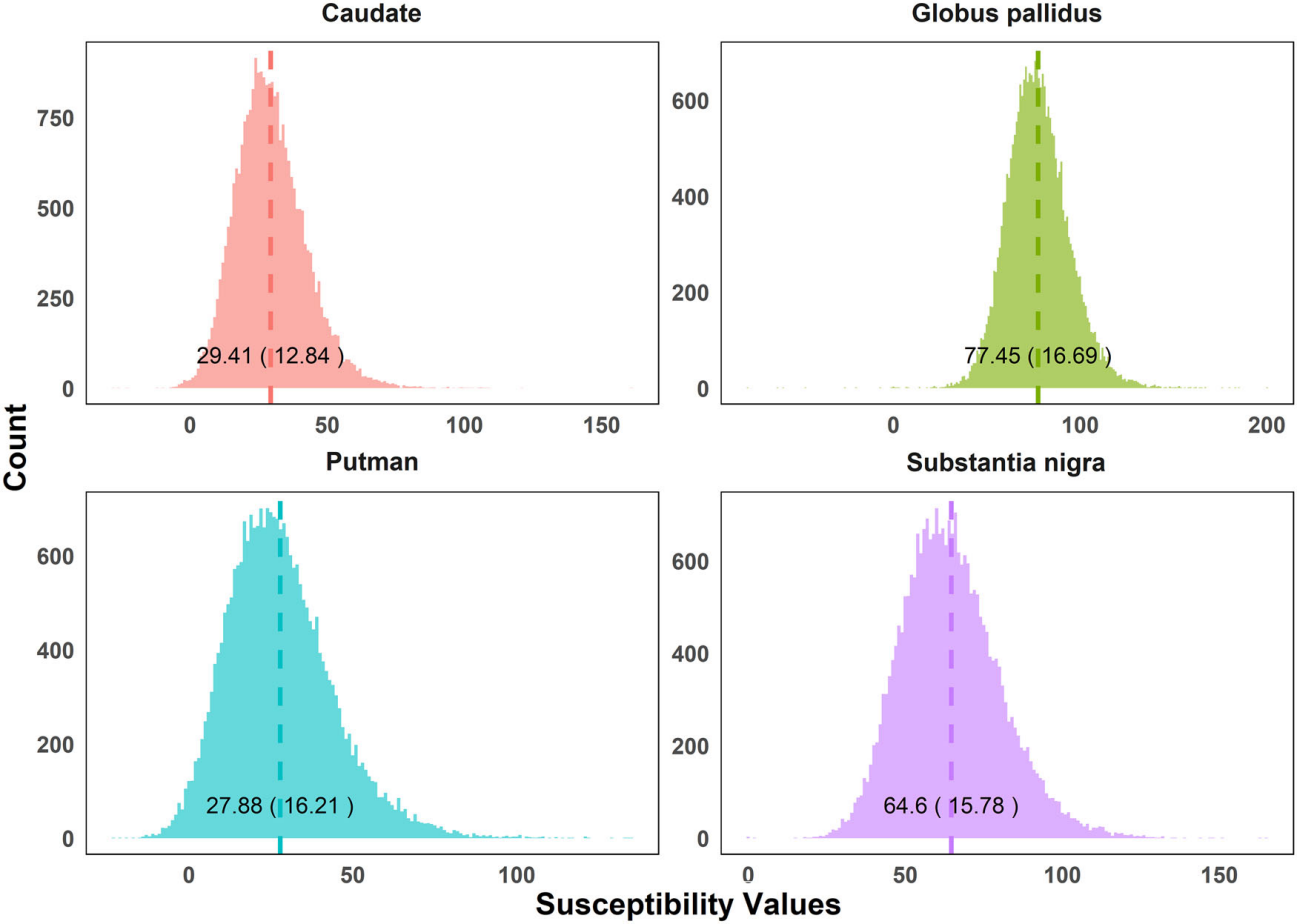
Distribution and mean (standard deviation) of susceptibility values for caudate, substantia nigra, globus pallidus, and putamen.

Table 1 summarizes the baseline characteristics of the 25,980 study participants, organized by demographic characteristics, lifestyle factors, and biological factors. Demographic characteristics showed a gender distribution of 54.4% female, an age range from 50 to 79 years (mean 65), and an ethnic composition with Whites constituting the vast majority (97.7% in females, 97.3% in males). Lifestyle factors revealed that 46.4% of participants consumed ≥ 4 cups of tea daily, while coffee consumption was more evenly distributed. Current smoking was reported by 2.7% of participants, with 33.3% being previous smokers. Biological factors including blood biomarkers and anthropometric measures are detailed in Table 1. Figure 1 displays the distribution and mean (standard deviation) of susceptibility values for all four basal ganglia nuclei.

**TABLE 1:** Baseline population characteristics of the 25,980 study participants in the UK Biobank, by sex.

Characteristics	Total (*N* = 25,980)	Female (*N* = 14,131)	Male (*N* = 11,849)	*p*
Sex, %, female	54.4			
Ethnicity, %			0.08	
White	97.5	97.7	97.3	
Asian	0.8	0.5	1.2	
Black	0.6	0.5	0.6	
Chinese	0.2	0.2	0.2	
Mixed	0.4	0.5	0.3	
Others	0.5	0.5	0.5	
Duration of moderate activity, min/day	65.53(64.40)	65.96(62.50)	65.03(66.51)	< 0.001
Age, years	65.04(7.34)	64.3(7.27)	65.91(7.34)	< 0.001
DBP, mmHg	78.62(10.64)	77.03(10.68)	80.51(10.29)	< 0.001
SBP, mmHg	141.44(20.15)	138.86(20.99)	144.49(18.64)	< 0.001
Waist circumference, cm	86.93(11.86)	81.72(10.90)	93.12(9.79)	< 0.001
BMI, kg/m^2^	26.01(3.93)	25.58(4.24) 26.53(3.47)	< 0.001	
Sleep duration, h/day	7.17(0.99)	7.11(0.992) 7.23(0.98)	< 0.001	
C‐reactive protein	1.92(3.39)	1.99(3.48)	1.83(3.28)	< 0.001
Lymphocyte count, 10^9^ cells/L	1.86(1.28)	1.92(0.89)	1.78(1.61)	< 0.001
Monocyte count, 10^9^ cells/L	0.38(0.20)	0.34(0.17)	0.43(0.22)	< 0.001
Neutrophil count, 10^9^ cells/L	4.28(1.32)	4.18(0.27)	4.39(1.36)	< 0.001
Platelet count, 10^9^ cells/L	231.59(53.30)	244.12(53.01)	216.97(49.89)	< 0.001
Coffee intake, cups/day			< 0.001	
0–1		45.3	48.8	41.2
2–3		37.1	35.5	39
≥ 4		17.5	15.6	19.8
Tea intake, cups/day				< 0.001
0–1		22.3	22.3	22.3
2–3		31.3	30.3	32.4
≥ 4		46.4	47.4	45.3
Alcohol intake frequency			< 0.001	
1		26.5	32	19.9
2		27.4	28.4	26.2
3		46.1	39.6	53.9
Insomnia				< 0.001
Never/rarely	22.8	16.8	30	
Sometimes	47.2	49.9	44	
Usually		30	33.3	26.1
Smoking status				< 0.001
Never		64	67	60.3
Previously		33.3	30.6	36.5
Current		2.7	2.4	3.2

*Note*: For alcohol intake frequency, those who self‐reported as “never,” “only on special occasions,” or “one to three times a month” were grouped together (Alcohol 1), those reporting “once or twice a week” formed a second group (Alcohol 2), and those reporting “three or four times a week, daily or almost daily” were categorized into a third group (Alcohol 3). For continuous variables, the values shown are mean (standard deviation); for categorical variables, the data are presented as percentages. *p*‐values are from the *t* test or chi‐square test comparing characteristics between men and women in the study cohort.

Abbreviations: BMI = body mass index; DBP = diastolic blood pressure; SBP = systolic blood pressure.

### 4.2 Associations With Basal Ganglia Susceptibility Values

#### 4.2.1 Demographic Characteristics

Analysis of demographic characteristics revealed significant associations with basal ganglia iron deposition (Table 2). Age demonstrated substantial positive effects on most basal ganglia regions, with the strongest association observed in the putamen (*β* = 0.313), followed by the caudate nucleus (*β* = 0.175) and globus pallidus (*β* = 0.069). No significant age effect was observed in the substantia nigra. Female sex showed a significant positive association only with substantia nigra susceptibility values (*β* = 0.076). Ethnic differences were notable, with Asian (−0.027), Black (−0.020), and other (−0.022) ethnicities showing significant negative associations with overall basal ganglia susceptibility compared to White participants (Table 3).

**TABLE 2:** Regression analysis with susceptibility values of caudate, putamen, globus pallidus, and substantia nigra as dependent variables.

Variables	Caudate		Putman		Pallidum		Substantia nigra	
	*β*	*p*	*β*	*p*	*β*	*p*	*β*	*p*
Age, years	0.18	< 0.001	0.31	< 0.001	0.07	< 0.001	0.02	0.08
Pulse pressure, mmHg	−0.01	< 0.001	−0.01	0.17	0	0.75	−0.02	0.05
C‐reactive protein, mg/L	0.03	< 0.001	0.01	0.4	0.05	< 0.001	0.03	< 0.001
Waist circumference, cm	0.12	< 0.001	0.12	< 0.001	0.06	< 0.001	0.01	0.15
Sex (baseline = male)	0.02	0.04	−0.02	0.05	0.02	0.1	0.08	< 0.001
Ethnicity (baseline = white)								
Asian	−0.04	< 0.001	−0.05	< 0.001	0.01	0.22	−0.01	0.09
Black	−0.04	< 0.001	−0.05	< 0.001	0.02	0.01	0	0.73
Chinese	−0.01	0.11	0	0.7	0.02	0.01	0.01	0.12
Mixed	0	0.69	−0.02	0.02	0.03	< 0.001	0.02	0.03
Others	−0.02	0.01	−0.03	< 0.001	−0.01	0.31	−0.02	0.02
Coffee intake (baseline = 0–1cups/day)								
2–3	0.01	0.19	0.01	0.49	0.01	0.11	0	0.85
≥ 4	0.02	0.03	0.02	0.07	0.01	0.21	0	0.64
Tea intake (baseline = 0–1cups/day)								
2–3	−0.04	< 0.001	−0.02	0.03	−0.03	< 0.001	−0.05	< 0.001
≥ 4	−0.06	< 0.001	−0.03	< 0.001	−0.06	< 0.001	−0.09	< 0.001
Alcohol intake frequency (baseline = Alcohol 1)								
Alcohol 2	0.01	0.22	0.02	0.06	−0.02	0.03	0.01	0.59
Alcohol 3	0.04	< 0.001	0.06	< 0.001	−0.04	< 0.001	0.02	0.08
Insomnia (baseline = never/rarely)								
Sometimes	0	0.79	−0.01	0.45	0.01	0.36	−0.01	0.63
Usually	0	0.64	0	0.74	0.02	0.05	−0.01	0.22
Smoking status (baseline = never)								
Previously	0.05	< 0.001	0.06	< 0.001	0.01	0.18	0.03	< 0.001
Current	0.05	< 0.001	0.06	< 0.001	0.06	< 0.001	0.06	< 0.001
Duration of moderate activity, min/day	−0.01	0.16	−0.01	0.13	0	0.62	0	0.58

*Note*: Pulse pressure = DBP − SBP. For alcohol intake frequency, those who self‐reported as “never,” “only on special occasions,” or “one to three times a month” were grouped together (Alcohol 1), those reporting “once or twice a week” formed a second group (Alcohol 2), and those reporting “three or four times a week, daily or almost daily” were categorized into a third group (Alcohol 3).

Abbreviations: DBP = diastolic blood pressure; SBP = systolic blood pressure.

**TABLE 3:** Regression analysis with average susceptibility value of the basal ganglia as outcome variable.

Variables	Basal ganglia				
	Unstandardized coefficient, *B*	Standard error, SE	Standardized coefficient, *β*	*t*	*p*
Age, years	0.29	0.01	0.18	21.93	< 0.001
Pulse pressure, mmHg	−0.01	0.01	−0.01	−1.26	0.21
C‐reactive protein, mg/L	1.72	0.37	0.04	4.64	< 0.001
Waist circumference, cm	0.1	0.01	0.1	10.78	< 0.001
Sex (baseline = man)	0.71	0.21	0.03	3.36	< 0.001
Ethnicity (baseline = white)					
Asian	−3.79	1.03	−0.03	−3.69	< 0.001
Black	−3.46	1.27	−0.02	−2.72	0.01
Chinese	1.77	2.03	0.01	0.87	0.38
Mixed	1.55	1.41	0.01	1.1	0.27
Others	−3.88	1.28	−0.02	−3.04	< 0.001
Coffee intake (baseline = 0–1cups/day)					
2–3	0.22	0.2	0.01	1.09	0.28
≥ 4	0.39	0.27	0.01	1.47	0.14
Tea intake (baseline = 0–1cups/day)					
2–3	−1.14	0.24	−0.05	−4.55	< 0.001
≥ 4	−1.92	0.21	−0.08	−7.97	< 0.001
Alcohol intake frequency (baseline = Alcohol 1)					
Alcohol 2	0.08	0.22	0	0.35	0.73
Alcohol 3	0.56	0.21	0.02	2.53	0.01
Insomnia (baseline = never/rarely)					
Sometimes	0	0.25	0	0.02	0.99
Usually	0.08	0.21	0	0.31	0.76
Smoking status (baseline = never)					
Previously	1.19	0.19	0.05	6.21	< 0.001
Current	5.48	0.56	0.07	9.84	< 0.001
Duration of moderate activity, min/day	0	0	−0.01	−1.22	0.22

*Note*: Pulse pressure = DBP − SBP. For alcohol intake frequency, those who self‐reported as “never,” “only on special occasions,” or “one to three times a month” were grouped together (Alcohol 1), those reporting “once or twice a week” formed a second group (Alcohol 2), and those reporting “three or four times a week, daily or almost daily” were categorized into a third group (Alcohol 3).

*p* < 0.05 shown in bold.

Abbreviations: DBP = diastolic blood pressure; SBP = systolic blood pressure.

#### 4.2.2 Lifestyle Factors

Tea consumption demonstrated dose‐dependent protective effects across basal ganglia regions. Daily consumption of ≥ 4 cups had significant negative impacts on all four nuclei (−0.063 in caudate, −0.032 in putamen, −0.063 in globus pallidus, −0.093 in substantia nigra). Consumption of 2–3 cups daily showed weaker but still significant negative associations with the caudate nucleus (−0.037), globus pallidus (−0.033), and substantia nigra (−0.048), but not the putamen.

Smoking status showed consistent positive associations with iron deposition. Current smoking was significantly associated with increased susceptibility values across all four basal ganglia nuclei (*β* = 0.053 in caudate, 0.057 in putamen, 0.057 in globus pallidus, 0.061 in substantia nigra). Previous smoking history also showed significant positive effects on the caudate nucleus (*β* = 0.046), putamen (*β* = 0.060), and substantia nigra (*β* = 0.032), but not the globus pallidus.

Coffee consumption showed no significant associations with susceptibility values in any of the four basal ganglia nuclei, despite adequate statistical power.

Alcohol intake frequency showed region‐specific effects. Higher frequency alcohol consumption (≥ 3 times weekly) had positive impacts on the caudate nucleus (*β* = 0.036) and putamen (*β* = 0.062) but a negative impact on the globus pallidus (*β* = −0.035). No significant effects were observed in the substantia nigra.

Physical activity (duration of moderate activity) and insomnia status showed no significant associations with basal ganglia susceptibility values.

#### 4.2.3 Biological Factors

Inflammatory markers showed significant associations with brain iron deposition. C‐reactive protein demonstrated positive correlations with susceptibility values in the caudate nucleus (*β* = 0.028), globus pallidus (*β* = 0.046), and substantia nigra (*β* = 0.031), but not in the putamen.

Anthropometric measures revealed strong associations with iron deposition. Waist circumference showed significant positive effects on the caudate nucleus (*β* = 0.115), putamen (*β* = 0.122), and globus pallidus (*β* = 0.058), with effect sizes second only to age among all measured factors.

Cardiovascular parameters showed limited associations. Pulse pressure (SBP minus DBP) did not significantly affect basal ganglia susceptibility values.

#### 4.2.4 Overall Basal Ganglia Susceptibility Analysis

In Table 3, we conducted regression analysis on the overall susceptibility value of the basal ganglia (average across all four nuclei). The findings largely corroborated the individual regional analyses, with age (*β* = 0.181), C‐reactive protein (*β* = 0.036), waist circumference (*β* = 0.097), female sex (*β* = 0.030), moderate tea consumption (*β* = −0.045), high tea consumption (*β* = −0.081), high alcohol intake frequency (*β* = 0.024), previous smoking (*β* = 0.047), and current smoking (*β* = 0.073) remaining significant factors. In this comprehensive analysis, ethnic differences became more apparent, with Asian, Black, and other ethnicities showing significant protective associations against iron deposition compared to White participants.

#### 4.2.5 Tea Consumption Dose‐Response Analysis

**FIGURE 2:**
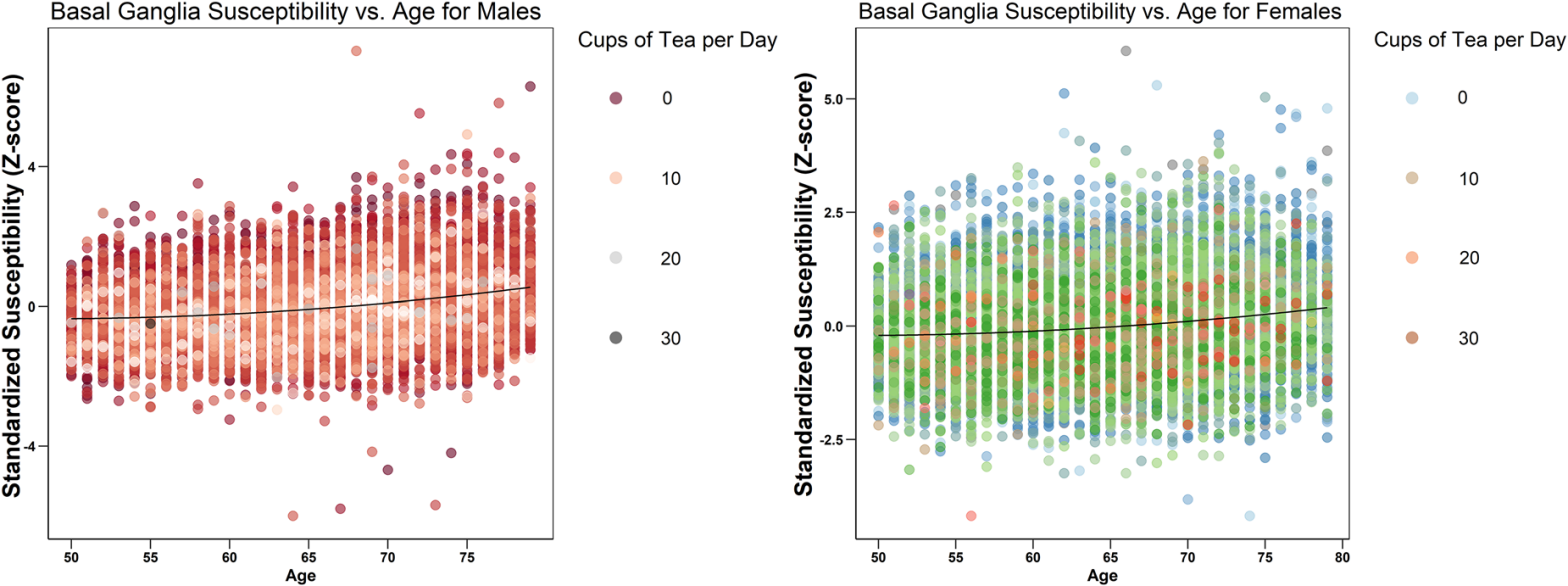
The scatter plot illustrates the relationship between the average standardized susceptibility values of the basal ganglia region (men on the left; women on the right) and age (*X*‐axis). The plot includes a LOWESS regression line. The legend on the right side of the scatter plot uses different colors to represent the number of cups of tea consumed daily.

**FIGURE 3:**
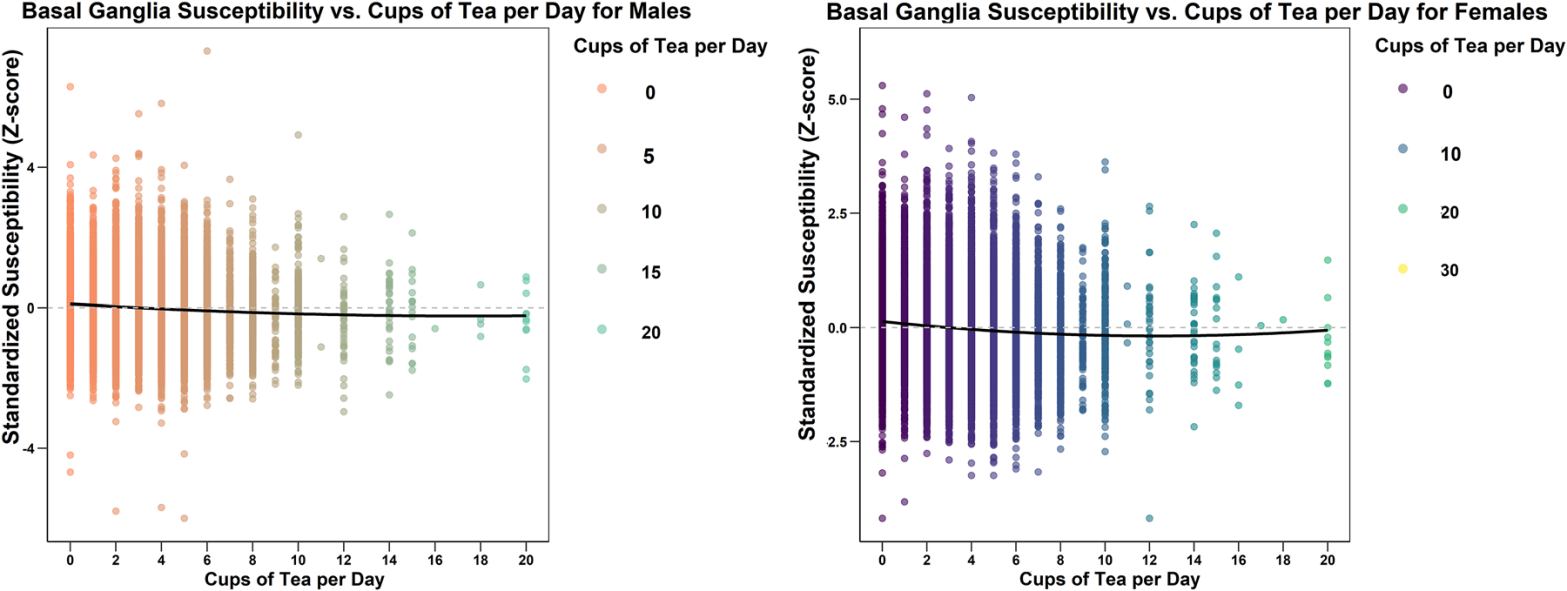
The scatter plot represents the relationship between the average standardized susceptibility values of the basal ganglia region (men on the left; women on the right) and the number of cups of tea consumed daily (*X*‐axis). The plot includes a LOWESS regression line. The legend on the right side of the scatter plot uses different colors to denote the number of cups of tea consumed daily.

The scatter plots in Figures 2 and 3 illustrate the relationship between the overall susceptibility value of the basal ganglia (standardized) with age and tea intake (in log scale), respectively. The relationship between age and the susceptibility value of the basal ganglia is observed to be positive, while the relationship between tea intake and the susceptibility value is negative. Polynomial regression lines included in each scatter plot indicate slight nonlinearities in the relationships, with the curves displaying a concave shape. Consequently, in our subsequent regression models, we have incorporated both the linear and quadratic terms for age and tea intake to account for these observed nonlinearities.

We further utilized our regression model to quantify the predicted change in the susceptibility values of the basal ganglia associated with an increase of three cups of tea intake per day while controlling for important variables such as daily coffee intake, insomnia status, smoking status, and BMI (Table 4). To reflect the impact of daily tea intake more intuitively on the brain, we compared the effect of tea drinking on the brain to the impact of rejuvenation on the brain in an average 65‐year‐old participant from the UK Biobank. Given the nonlinear relationship between susceptibility value and tea intake, the associations vary across the consuming range. Based on our linear regression, we calculated that in males, increasing the daily tea consumption from zero to three cups would reduce the susceptibility value of the basal ganglia by 0.044 standard deviations, equivalent to a “rejuvenation” effect of 1.50 years on the brain. In females, this increase would lead to a 0.162 standard deviation decrease in the susceptibility value of the basal ganglia, equivalent to a “rejuvenation” effect of 5.59 years on the brain. For males, increasing daily tea consumption from three to three cups is equivalent to a “rejuvenation” effect of 1.6 years on the brain, while for females, this effect is equivalent to 3.42 years. Increasing daily tea consumption from six to nine cups is equivalent to a “rejuvenation” effect of 1.39 years for males and 2.63 years for females. The effect diminishes to 1.24 years for males and 1.50 years for females when increasing tea consumption from 9 to 12 cups (Table 5). Regardless of the interval of daily tea intake, the impact of tea on the susceptibility value of the basal ganglia is always greater in females than in males.

**TABLE 4:** Regression analysis with the average susceptibility value of the basal ganglia as the dependent variable.

Variables	Basal ganglia				
	*B*	SE	*β*	*t*	*p*
Intake	−0.1	0.01	−0.1	−11.91	< 0.001
Intake^2^	−0.01	0.01	−0.01	−1.42	0.16
Intake × male	0.06	0.01	0.06	8.38	< 0.001
Intake × std. age	−0.04	0.02	−0.02	−1.78	0.07
Std. age	0.22	0.01	0.22	16.37	< 0.001
Std. age^2^	0.04	0.01	0.04	7	< 0.001

*Note*: Intake is measured in log(1 + daily cups of tea).

Abbreviations: Intake = cups of tea per day; Std. age = standard age; *B* = unstandardized coefficient; SE = standard error; *β* = standardized coefficient.

**TABLE 5:** Predicted equivalent effect of rejuvenation in terms of decreasing years for an average 65‐year‐old individual.

Tea intake (drinks per day)	Male		Female	
	Susceptibility value	Equivalent rejuvenation at 65	Susceptibility value	Equivalent rejuvenation at 65
0–3	−0.04	1.50 years	−0.16	5.59 years
3–6	−0.04	1.60 years	−0.09	3.42 years
6–9	−0.03	1.39 years	−0.07	2.63 years
9–12	−0.03	1.24 years	−0.05	2.21 years

To further explore potential gender‐specific patterns, stratified scatter plots for coffee consumption, C‐reactive protein, and waist circumference are provided in the Supporting Information (Figures S1–S3). Detailed regression results across all four basal ganglia nuclei, including Bonferroni‐adjusted *p*‐values, are provided in Table S1.

## 5 Discussion

Our study investigates the impact of lifestyle factors and demographic indicators on the susceptibility value of the basal ganglia. We included 25,980 participants from one of the largest recent cohorts, controlling for significant confounders, which enhances the statistical power and credibility of our findings. Similar to previous studies, age, waist circumference, smoking, and gender have significant positive correlations with the health of the basal ganglia gray matter nuclei (Almeida et al. 2008; Verstynen et al. 2012; Janowitz et al. 2015; Cherubini et al. 2009). Studies have shown that brain inflammation can lead to iron metabolism disorders, thereby affecting the susceptibility value of the basal ganglia nuclei (Ward et al. 2014; Mayer et al. 2023). Our findings indicate a significant correlation between the peripheral inflammation marker (C‐reactive protein) and increased susceptibility values of the caudate nucleus, globus pallidus, and substantia nigra. Moreover, changes in SBP and DBP can damage vascular endothelial cells, disrupt the blood–brain barrier, and lead to iron metabolism disorders affecting the susceptibility value of the gray matter nuclei (Chistiakov et al. 2015; Santisteban et al. 2020; P. Wang, Ren, et al. 2022). However, our further research found that pulse pressure (systolic − diastolic) does not significantly affect the susceptibility value of the basal ganglia. Asians, Blacks, and other races showed a significant association with decreased overall susceptibility values of the caudate nucleus, globus pallidus, substantia nigra, and putamen. Moreover, we found that tea consumption was significantly associated with a decrease in the susceptibility value of almost all basal nuclei.

Consuming ≥ 4 cups of tea per day compared to 0–1 cups can reduce the susceptibility value in the caudate nucleus (−0.063 change in SD per SD change in basal caudate), globus pallidus (−0.063 in basal pallidum), putamen (−0.032 in basal putamen), and substantia nigra (−0.093 in nigra). In the group consuming two to three cups of tea daily, there is also a significant negative association with the susceptibility values of the caudate nucleus, globus pallidus, and substantia nigra, but this association is weaker than in the group consuming four or more cups of tea daily, indicating that increased daily tea consumption provides stronger protection for the basal ganglia nuclei. The types of tea analyzed were green tea and black tea, which contain different bioactive components due to their distinct processing methods. Green tea contains catechins (especially EGCG) with potent antioxidant properties, while black tea contains theaflavins and thearubigins, which have different antioxidant characteristics due to the conversion of catechins during fermentation (Tipoe et al. 2007; Truong and Jeong 2021). These components can act as chelators and antioxidants for iron, helping to reduce free radical damage, thereby potentially lowering brain iron deposition and associated oxidative stress (Weinreb et al. 2004). The components in green and black tea may offer neuroprotective effects through inhibiting the formation of neurodegenerative disease‐related proteins such as Aβ (Bieschke et al. 2010). EGCG in green tea has shown protective effects on the nervous system in models of AD and Parkinson's disease (Levites et al. 2001). Our study indicates that the neuroprotective effects of tea (green or black) still exist in a healthy middle‐aged and elderly population. Furthermore, the impact of tea on the overall susceptibility value of the basal ganglia shows gender differences, with stronger effects in females. This sensitivity is particularly evident in those who consume 0–3 cups of tea daily. Gender differences may be related to hormone levels, metabolic differences, and variations in lifestyle and dietary habits, which could affect the absorption, metabolism, and brain impact of components in black and green tea (Morand et al. 2020).

In our study, no significant correlation was observed between coffee intake and the susceptibility values of the caudate nucleus, putamen, globus pallidus, and substantia nigra. However, other research indicates that higher coffee consumption may help reduce the deposition of amyloid proteins in the brain (J. W. Kim et al. 2019). Furthermore, coffee intake has been positively associated with white matter integrity and cortical thickness, and in males, a negative correlation has been found between coffee consumption and the incidence of cerebral hemorrhage (Mayer et al. 2023; Shinoda et al. 2015). These factors, such as controlling neuroinflammation, maintaining white matter integrity, and reducing cerebral microbleeds, seem to contribute to maintaining iron homeostasis. However, when considering a large sample size of several thousand individuals and accounting for other significant confounders, no significant association was found between coffee intake and the susceptibility value of the basal ganglia in our study.

Smoking leads to the release of reactive oxygen species (ROS), increasing oxidative stress and the production of inflammatory mediators (Valavanidis et al. 2013; Pryor and Stone 1993). This results in endothelial cell damage and dysfunction (Celermajer et al. 1993), which in turn can trigger brain inflammation and blood–brain barrier damage (Paulson et al. 2010). These processes can further exacerbate iron metabolism disorders in the brain, leading to increased susceptibility values in the brain nuclei of smokers (Gao et al. 2025). Previous studies have shown that smoking can cause an increase in the susceptibility value of the thalamus (J. Li, Zhang, et al. 2021). In individuals who previously smoked, there is a significant positive association between smoking and the susceptibility values of the caudate nucleus, putamen, and substantia nigra. In current smokers, smoking is significantly positively associated with the susceptibility values of the caudate nucleus, putamen, globus pallidus, and substantia nigra.

A study on a healthy population aged 21–58 found a linear correlation between age and the basal ganglia nuclei (caudate nucleus, globus pallidus, substantia nigra, and putamen) (Burgetova et al. 2021). However, our study did not observe a significant correlation between age and the substantia nigra, which might be attributed to the age differences in our study population. Our participants, aged between 60 and 79 years, represent an older demographic compared to the 21–58 age range, and this age group might exhibit different patterns of susceptibility value changes. Our large sample size provides adequate statistical power to detect associations in this specific age range, complementing findings from studies in younger populations.

Neuroinflammation and iron dysregulation are considered significant mechanisms in AD, mutually reinforcing each other (Long et al. 2022; Liu et al. 2022; Ayton et al. 2015). Iron metabolism disorders can alter the inflammatory phenotype of microglia, and conversely, neuroinflammation can exacerbate iron metabolism disorders. CRP (C‐reactive protein) levels, as an indicator of peripheral inflammation, may reflect the state of neuroinflammation in the brain, potentially affecting microglial activity and iron accumulation (Koyama et al. 2013). Genes associated with microglia and AD progression, including those related to immune responses (such as CR1, CD33) (Hollingworth et al. 2011), suggest that CRP‐mediated neuroinflammation could promote disease pathology by influencing microglial function (Villegas‐Llerena et al. 2016). Thus, it is believed that there is an association between C‐reactive protein, brain inflammation, iron metabolism disorders, and neurodegenerative diseases. Our study found that CRP has a significant positive correlation with iron deposition in the caudate nucleus, globus pallidus, and substantia nigra. This effect is also found in the healthy middle‐aged and elderly population, providing important insights for our early preventive strategies.

Previous studies have indicated that obesity can lead to neurovascular uncoupling and disrupt the blood–brain barrier, thereby activating neuroinflammatory processes and triggering iron metabolism disorders (Gustafson et al. 2007). This process is further exacerbated by aging (Tarantini et al. 2018; Tucsek et al. 2014; Valcarcel‐Ares et al. 2019). Moreover, further studies have shown that changes in waist circumference are associated with brain iron deposition in the striatum, amygdala, and hippocampus, and are also related to circulating Aβ42 levels (Blasco et al. 2017). The accumulation of Aβ42 can activate microglia and astrocytes, leading to the release of inflammatory cytokines, further exacerbating iron metabolism disorders (McCarthy et al. 2018). Our research found a significant positive correlation between waist circumference and the susceptibility of the caudate nucleus, putamen, and globus pallidus. The positive effect of waist circumference on the susceptibility values of these nuclei is second only to aging.

In the regression analysis of the total susceptibility value of all four nuclei, it was found that the basal ganglia susceptibility value in Black individuals is lower than in White individuals (−0.020 change in SD per SD change in basal ganglia), and this value shifts to −0.027 in Asian individuals. This suggests that the basal ganglia nuclei of Asians and Blacks may have a stronger regulatory capacity against iron deposition caused by various factors.

The current study has several potential limitations. Considering the nature of volunteers and their relatively younger age at recruitment, the study participants might represent a healthier segment of the UK population. However, our research focuses on this relatively healthy population, which does not impact our findings. Moreover, we have explored the relationship between various demographic indicators and lifestyle factors and the susceptibility values of the basal ganglia nuclei. Yet, given the inherent limitations of an observational design, we cannot rule out the possibility of residual confounding or bias due to unknown or unmeasured factors. Furthermore, some data collection was conducted through questionnaires, which may introduce recall bias. Lastly, our study relies on a cross‐sectional design, which does not allow for the identification of causal relationships. Although our model accounted for many potential confounders, we cannot exclude the possibility of reverse causality.

## 6 Conclusions

In summary, this study is the largest to date that investigates the relationship between the susceptibility values of basal ganglia nuclei and demographic indicators as well as lifestyle factors. It not only corroborates many known associations but also reveals previously unidentified connections, such as the relationship between lifestyle factors like tea consumption and the susceptibility values of basal ganglia nuclei. We provide evidence for a negative correlation between tea intake and iron deposition in the basal ganglia nuclei among the generally healthy middle‐aged and elderly population.

## Author Contributions

P.L. and L.G. conceived and designed the study. P.L. and C.L. analyzed data. P.L., L.Y., and L.G. interpreted data. P.L. and L.G. wrote the paper. L.G., C.L., and L.Y. critically edited the work. All authors approved the final version to be submitted for publication and agree to be accountable for all aspects of this work.

## Ethics Statement

The UK Biobank protocol was approved by the NHS North West Multicentre Research Ethics Committee (21/NW/0157).

## Consent

All participants provided informed consent at recruitment, allowing for follow‐up using data linkage to health records.

## Conflicts of Interest

The authors declare no conflicts of interest.

## Peer Review

The peer review history for this article is available at https://publons.com/publon/10.1002/brb3.70862

## Supporting information



Table S1. Regression analysis with susceptibility values of caudate, putamen, globus pallidus, and substantia nigra as dependent variables
Figure S1. Sex‐stratified associations between coffee consumption and basal ganglia susceptibility values.
Figure S2. Sex‐stratified associations between C‐reactive protein (CRP) and basal ganglia susceptibility values.
Figure S3. Sex‐stratified associations between waist circumference and basal ganglia susceptibility values.

## Data Availability

Data and materials are available via UK Biobank at http://www.ukbiobank.ac.uk/.
