# Supplementary material for: Multiple Demographic, Lifestyle, and Biological Factors Associated With Brain Iron Deposition in the Basal Ganglia: A Comprehensive Analysis of 25,980 UK Biobank Participants
Source: Brain Behav. 2025 Sep 10;15(9):e70862. doi: 10.1002/brb3.70862 (PMC12423436; doi:10.1002/brb3.70862)
Supplement: Supplementary file 1 — Table S1. Regression analysis with susceptibility values of caudate, putamen, globus pallidus, and substantia nigra as dependent variables Figure S1. Sex‐stratified associations between coffee consumption and basal ganglia susceptibility values. Figure S2. Sex‐stratified associations between C‐reactive protein (CRP) and basal ganglia susceptibility values. Figure S3. Sex‐stratified associations between waist circumference and basal ganglia susceptibility values. [file BRB3-15-e70862-s001.docx]

**Supplementary Material**

| **Table S1. Regression analysis with susceptibility values of caudate, putamen, globus pallidus, and substantia nigra as dependent variables** | | | | | | | | | | | | |
| --- | --- | --- | --- | --- | --- | --- | --- | --- | --- | --- | --- | --- |
|  |  |  |  |  |  |  |  |  |  |  |  |  |
| **Variables** | **Caudate** | | | **Putamen** | | | **Pallidum** | | | **Substantia Nigra** | | |
|  |  |  |  |  |  |  |  |  |  |  |  |  |
|  | **β** | **P** | **P*** | **β** | **P** | **P*** | **β** | **P** | **P*** | **β** | **P** | **P*** |
| Age, years | 0.18 | <0.001 | <0.001 | 0.31 | <0.001 | <0.001 | 0.07 | <0.001 | <0.001 | 0.02 | 0.07 | 0.28 |
| Pulse pressure, mmHg | -0.01 | <0.001 | <0.001 | -0.01 | 0.17 | 0.68 | 0 | 0.75 | >0.99 | -0.02 | 0.05 | 0.2 |
| C-reactive protein, mg/L | 0.03 | <0.001 | <0.001 | 0.01 | 0.39 | >0.99 | 0.05 | <0.001 | <0.001 | 0.03 | <0.001 | <0.001 |
| Waist circumference, cm | 0.12 | <0.001 | <0.001 | 0.12 | <0.001 | <0.001 | 0.06 | <0.001 | <0.001 | 0.01 | 0.15 | 0.6 |
| Sex (baseline = male) | 0.02 | 0.04 | 0.16 | -0.02 | 0.06 | 0.24 | 0.02 | 0.1 | 0.4 | 0.08 | <0.001 | <0.001 |
| **Ethnicity (baseline = white)** | | | | | | | | | | | | |
|  |  |  |  |  |  |  |  |  |  |  |  |  |
| Asian | -0.04 | <0.001 | <0.001 | -0.05 | <0.001 | <0.001 | 0.02 | 0.01 | 0.04 | 0 | 0.73 | >0.99 |
| Black | -0.01 | 0.11 | 0.44 | -0.02 | 0.02 | 0.08 | 0.03 | <0.001 | <0.001 | -0.02 | 0.09 | 0.36 |
| Chinese | 0 | 0.68 | >0.99 | -0.02 | 0.02 | 0.1 | 0.03 | 0.001 | 0.004 | 0.02 | 0.03 | 0.12 |
| Mixed | -0.05 | <0.001 | <0.001 | -0.05 | <0.001 | <0.001 | 0.01 | 0.22 | 0.88 | -0.01 | 0.09 | 0.36 |
| Others | -0.02 | 0.01 | 0.04 | -0.03 | <0.001 | <0.001 | -0.01 | 0.31 | >0.99 | -0.02 | 0.02 | 0.08 |
| **Coffee intake (baseline = 0-1 cups/day)** | | | | | | | | | | | | |
| 2-3 cups/day | 0.01 | 0.19 | 0.76 | 0.01 | 0.5 | >0.99 | 0.01 | 0.11 | 0.44 | 0 | 0.85 | >0.99 |
| ≥4 cups/day | 0.02 | 0.03 | 0.12 | 0.02 | 0.07 | 0.28 | 0.01 | 0.21 | 0.84 | 0 | 0.64 | >0.99 |
| **Tea intake (baseline = 0-1 cups/day)** | | | | | | | | | | | | |
| 2-3 cups/day | -0.04 | <0.001 | <0.001 | -0.02 | 0.03 | 0.12 | -0.03 | 0.001 | 0.004 | -0.05 | <0.001 | <0.001 |
| ≥4 cups/day | -0.06 | <0.001 | <0.001 | -0.03 | 0.001 | 0.004 | -0.06 | <0.001 | <0.001 | -0.09 | <0.001 | <0.001 |
| **Alcohol intake frequency (baseline = Alcohol 1)** | | | | | | | | | | | | |
| Alcohol 2 | 0.01 | 0.22 | 0.88 | 0.02 | 0.06 | 0.24 | -0.02 | 0.03 | 0.12 | 0.01 | 0.59 | >0.99 |
| Alcohol 3 | 0.04 | <0.001 | <0.001 | 0.06 | <0.001 | <0.001 | -0.04 | <0.001 | <0.001 | 0.02 | 0.08 | 0.32 |
| **Insomnia (baseline = never/rarely)** | | | | | | | | | | | | |
| Sometimes | 0 | 0.77 | >0.99 | -0.01 | 0.48 | >0.99 | 0.01 | 0.35 | >0.99 | -0.01 | 0.63 | >0.99 |
| Usually | 0 | 0.62 | >0.99 | 0 | 0.77 | >0.99 | 0.02 | 0.05 | 0.2 | -0.01 | 0.22 | 0.88 |
| **Smoking status (baseline = never)** | | | | | | | | | | | | |
| Previously | 0.05 | <0.001 | <0.001 | 0.06 | <0.001 | <0.001 | 0.01 | 0.18 | 0.72 | 0.03 | <0.001 | <0.001 |
| Current | 0.05 | <0.001 | <0.001 | 0.06 | <0.001 | <0.001 | 0.06 | <0.001 | <0.001 | 0.06 | <0.001 | <0.001 |
| Duration of moderate activity, minutes/day | -0.01 | 0.17 | 0.68 | -0.01 | 0.14 | 0.56 | 0 | 0.62 | >0.99 | 0 | 0.58 | >0.99 |
| P* = Bonferroni-corrected p-values for multiple testing across four brain regions (correction factor = 4). Adjusted significance threshold: p < 0.0125. | | | | | | | | | | | | |
|  |  |  |  |  |  |  |  |  |  |  |  |  |
|  |  |  |  |  |  |  |  |  |  |  |  |  |
|  |  |  |  |  |  |  |  |  |  |  |  |  |


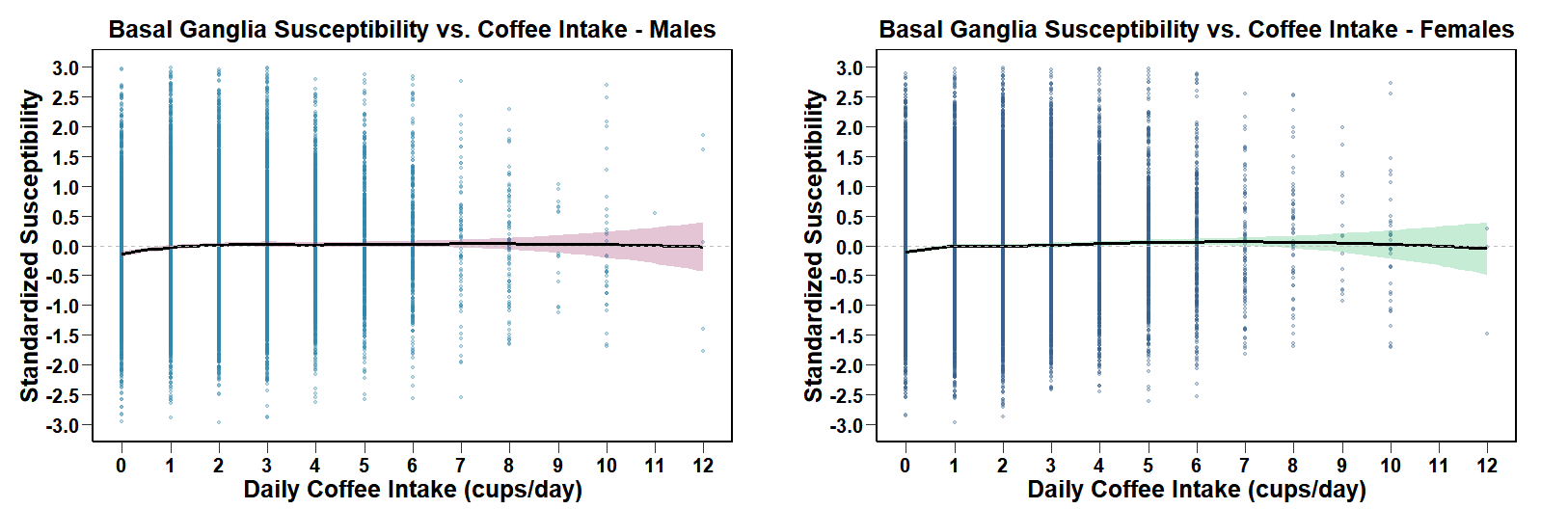


**Figure S1**. Sex-stratified associations between coffee consumption and basal ganglia susceptibility values.

Scatter plots show the relationship between daily coffee intake (cups/day) and overall basal ganglia susceptibility values in males (left) and females (right). Each dot represents an individual participant, and a LOWESS regression line is shown to illustrate the trend.


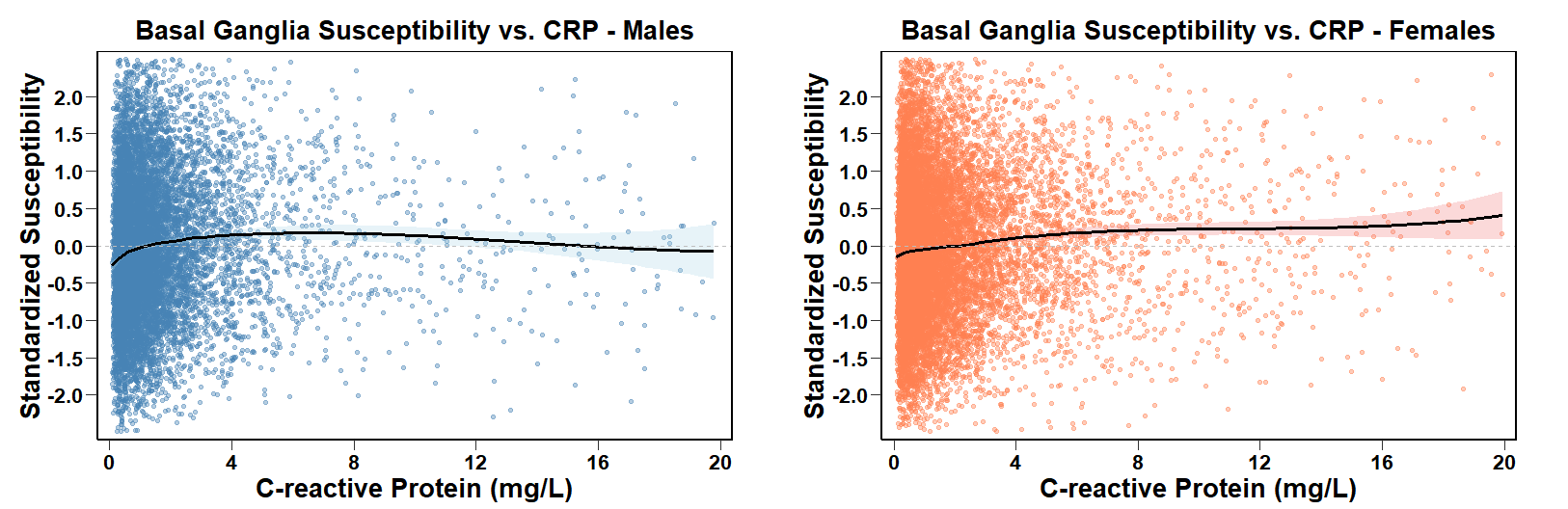


**Figure S2**. Sex-stratified associations between C-reactive protein (CRP) and basal ganglia susceptibility values.

Scatter plots illustrate the relationship between serum CRP levels and overall basal ganglia susceptibility values in males (left) and females (right). LOWESS regression lines are overlaid to visualize the trend.


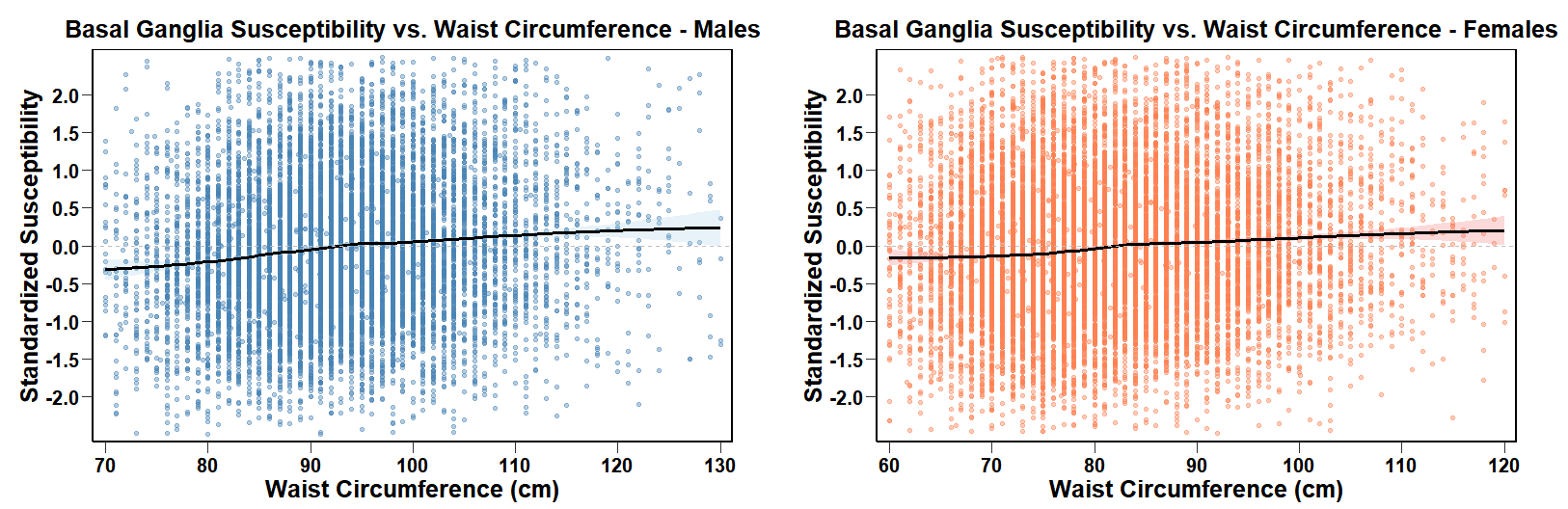


**Figure S3**. Sex-stratified associations between waist circumference and basal ganglia susceptibility values.

Scatter plots depict the relationship between waist circumference (cm) and overall basal ganglia susceptibility values in males (left) and females (right), with LOWESS regression lines indicating the trend.
